# Comparison and Evaluation of Integrity Algorithms for Vehicle Dynamic State Estimation in Different Scenarios for an Application in Automated Driving

**DOI:** 10.3390/s21041458

**Published:** 2021-02-19

**Authors:** Grischa Gottschalg, Stefan Leinen

**Affiliations:** Chair of Physical and Satellite Geodesy, Institute of Geodesy, Technical University of Darmstadt, Franziska-Braun-Straße 7, 64287 Darmstadt, Germany; leinen@psg.tu-darmstadt.de

**Keywords:** integrity, vehicle dynamic state estimation, GNSS, IMU, odometry, automated driving, sensor data fusion, Kalman filter, RAIM

## Abstract

High-integrity information about the vehicle’s dynamic state, including position and heading (yaw angle), is required in order to implement automated driving functions. In this work, a comparison of three integrity algorithms for the vehicle dynamic state estimation of a research vehicle for an application in automated driving is presented. Requirements for this application are derived from the literature. All implemented integrity algorithms output a protection level for the position and heading solution. In the comparison, four measurement data sets obtained for the vehicle dynamic state estimation, which is based on a Global Navigation Satellite Signal receiver, inertial measurement units and odometry information (wheel speeds and steering angles), are used. The data sets represent four driving scenarios with different environmental conditions, especially regarding the satellite signal reception. All in all, the Kalman Integrated Protection Level demonstrated the best performance out of the three implemented integrity algorithms. Its protection level bounds the position error within the specified integrity risk in all four chosen scenarios. For the heading error, this also holds true, with a slight exception in the very challenging urban scenario.

## 1. Introduction

Automated driving offers great potential to improve future transportation. The expected benefits include reduced fuel consumption, improved passenger safety, optimized traffic flow and enhanced mobility for people who are unable or unwilling to drive [1,2]. Therefore, many research projects are motivated by these expected benefits [3,4,5]. Even though every project has its own system architecture, eventually, in all projects, there is a need to estimate the vehicle’s dynamic state (including position and attitude) for subsequent tasks, e.g., the trajectory control. Different approaches for this estimation task can be found in the literature (e.g., [6,7]), which most often include several sensors, e.g., Global Navigation Satellite System (GNSS) receivers Inertial Measurement Units (IMU), sensors to measure wheel speeds and steering angels (odometry), Light Detection and Ranging (LiDAR) and Radio Detection and Ranging (RADAR) sensors, as well as cameras. Typically, filter (e.g., Kalman filter) or snapshot methods (e.g., least squares methods) are used for the sensor data fusion. In order to quantify the performance of such a navigation system, the quality parameters accuracy, integrity, continuity and availability are proposed by Pullen [8]. While there are many studies about the accuracy of navigation systems (e.g., [9]), which partly also take availability and continuity into account, up to now, integrity did not receive the appropriate attention in the automotive industry [10]. Integrity is crucial for automated driving, since it refers to the amount of trust one can place in the navigation system’s solution and its ability to output timely warnings when not to use it [11]. Consequently, to develop and implement automated driving functions of Level 3–5 of the SAE Standard J3016 [12], high-integrity information about the vehicle’s dynamic state will be necessary [13]. Therefore, there is an urgent need for integrity concepts for sensor data fusion algorithms to estimate the vehicle’s dynamic state which motivates the investigation presented here as part of our research project UNICAR*agil*.

This work describes further developments of integrity concepts that we previously presented in a conference paper [14]. Therefore, the same foundation, including the motivation, structure, definitions and requirements, as well as parts of the literature review and implementation, is used in this work.

In the collaborative project UNICAR*agil* carried out by a consortium of eight German universities and eight industrial partners, disruptive modular structures for agile, automated vehicle concepts are developed, and over a period of four years, four prototype vehicles of different characteristics are built [15]. The Vehicle Dynamic State Estimation (VDSE) has to fulfill high performance requirements regarding the mentioned parameters: accuracy, integrity, continuity and availability. Therefore, the localization algorithm to be developed does not only have to be accurate, precise and of high availability, it also has to provide integrity information about the estimated quantities [15]. The sensor setup of the VDSE in UNICAR*agil* includes a multi-constellation dual-frequency Real-Time Kinematic (RTK) GNSS receiver, two IMUs, which are both Micro-Electro-Mechanical Systems (MEMS) (one commercial grade MEMS-IMU and one mid-performance MEMS-IMU), and odometry sensors, which measure wheel speeds and steering angles. Detailed information about the used sensors is given in Section 4.1.

Besides the VDSE, there is a second sensor data fusion algorithm for localization in UNICAR*agil*, which is using a high-definition map and cameras to estimate the vehicle’s pose. The architecture describing the interaction between the two localization functions is explained in detail by Homolla et al. [16]. The actual concept of the VDSE contains several sensor fusion algorithms, which are developed by independent teams to reduce the risk of simultaneous error occurrences stemming from hidden design flaws [17]. The algorithms are utilizing different subsets of the available sensor information [17]. As an example for the comparison in this work, one of these algorithms is chosen which performs a loose coupling of GNSS and the mid-performance MEMS-IMU and is using the odometry data in addition.

In the remainder of this work, in Section 2, the definition of integrity monitoring, as well as the use case and requirements of the VDSE, are discussed. Afterwards, integrity concepts from the literature are reviewed in Section 3. The implementation of the sensor data fusion algorithm and the integrity algorithms is explained in Section 4. In Section 5, the carried out experiments and results are discussed. A conclusion and an outlook of future work are given in Section 6.

## 2. Definitions and Requirements

Before the actual integrity concepts are discussed, it is important to clarify what is meant by integrity and which requirements need to be fulfilled for the use case of the integrity concepts. These topics are covered by the following two Subsections.

### 2.1. Definitions

The definition of integrity is given by the Federal Radio Navigation Plan of the United States Government [11] as: “Integrity is the measure of the trust that can be placed in the correctness of the information supplied by a navigation system. Integrity includes the ability of the system to provide timely warnings to users when the system should not be used for navigation.” In order to further specify integrity, the following parameters are used, which are explained in the European Space Agency’s online encyclopedia [18], exemplary for the Position Error (PE):

- Alert Limit (AL): “The alert limit for a given parameter measurement is the error tolerance not to be exceeded without issuing an alert.”
- Time to Alert (TTA): “The maximum allowable time elapsed from the onset of the navigation system being out of tolerance until the equipment enunciates the alert.”
- Integrity Risk (IR): “Probability that, at any moment, the position error exceeds the Alert Limit.”
- Protection Level (PL): “Statistical bound error computed so as to guarantee that the probability of the absolute position error exceeding said number is smaller than or equal to the target integrity risk.”

**Figure 1:**
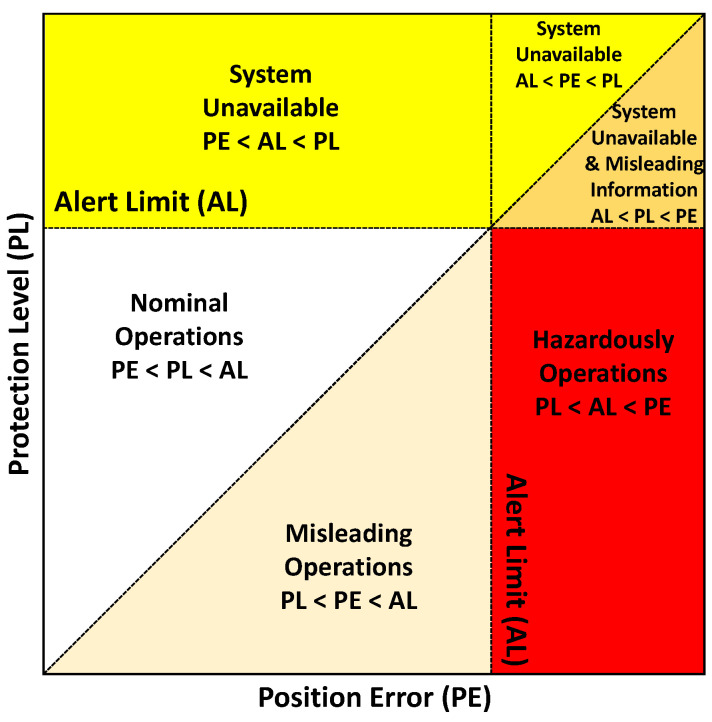
Stanford integrity diagram (Figure based on [18]).

A common way to visualize these parameters is the Stanford integrity diagram. Figure 1 shows such a diagram exemplary for the PE.

As long as the integrity algorithm works as intended, the PE is bound by the PL, which is represented by the area above the diagonal in the diagram. During normal operations, the PL is lower then the AL. If the PL exceeds the AL, the system becomes unavailable. In case of a PE greater than the PL, the integrity information is misleading, which is represented by the area under the diagonal in the diagram. This becomes a hazard if the PE is actually greater than the AL, which is the case in the red area in the diagram.

In general, integrity monitoring systems fulfill two tasks: different levels of fault detection and mitigation (including fault detection, recovery, isolation and/or exclusion) and solution protection ([19] chapter 17). We assume that the fault detection and mitigation is executed before the integrity level and therefore the integrity algorithms in this work only refer to solution protection.

### 2.2. Use Case and Requirements

When comparing different integrity concepts, it is important to keep the intended use case in mind. In this work, integrity algorithms for the fusion algorithms of the VDSE in UNICAR*agil* are compared. This leads to several requirements:

First, there are general requirements of the VDSE in UNICAR*agil*. These include that the outputs of the VDSE are available in real-time to be processed by other services in the automated vehicle, especially by the motion controller [15]. Additionally, a distinct statement about the quantities estimated by the VDSE is required, which includes a level of confidence, e.g., a PL ([20] chapter 5). Eventually, the VDSE will provide integrity information for all its outputs. However, in this work, only the horizontal position and the yaw estimate are considered, since they are crucial for collision avoidance in road traffic. Even though integrity information can be represented in many different forms, in this study, the comparison of a predefined, constant AL with a PL outputted by the integrity algorithm is preferred, which is consistent with the previously introduced Stanford integrity diagram.

Secondly, requirements for the integrity information in particular have to be considered, which stem from the use case of the VDSE. In order to define the required ALs of localization algorithms for an application in automated driving, one has to define the PL and the corresponding IR first. In the literature, 1-D and 2-D PLs are used with different assumptions about the road geometry and the acceptable IR. On one hand, Reid et al. [21] derive for different types of vehicles and types of roads in the US ALs using a 1-D PLs and an IR of $10^{-8}/h$: For a passenger vehicle on US local roads, the most similar scenario to the given use case in UNICAR*agil*, the ALs is given as 0.29 m in lateral and longitudinal direction of the vehicle, as 1.40 m in vertical direction and as $1.50{}^{\circ }$ for the attitude angles (roll, pitch, yaw). On the other hand, the European GNSS Agency (GSA) reports their requirements [22] for automated driving which include accuracy requirements, meaning 95%-quantile, for the localization algorithm of 20 cm horizontally (2-D) and 2 m, vertically, as well as integrity requirements in terms of ALs of 10 to 15 m with an integrity risk of $10^{-7}$. As these two examples show, there are no common integrity requirements for automated driving so far. Besides that, the accuracy requirements are still a challenge for localization algorithms developed up to now for an application in automated driving [21]. For the comparison of the implemented integrity algorithms in this study, a working hypothesis of 0.6 m as AL for the 2-D horizontal PE and $1.0{}^{\circ }$ as AL for the yaw angle or Heading Error (HE) with an IR of $10^{-2}$ is used. The ALs are chosen as an intermediate of the values from the literature mentioned before. The IR is set as less restrictive than the mentioned values, which are inspired by aviation applications, since these values are at the moment not realistic for GNSS-based VDSE fusion algorithms in the application of automated driving in urban areas.

## 3. State of the Art

In the literature, a great variety of algorithms to evaluate a system’s integrity can be found that stem from different research areas [23,24]. Since the application in this work is in VDSE or localization functions, we focus in the following on approaches from the domain of navigation, especially localization functions based on a sensor fusion using a GNSS receiver, and adopt the nomenclature from this domain. In order to differentiate the integrity concepts from the literature, we take certain properties of the localization function itself into account, since the integrity algorithms are typically developed for a specific system setup. In the following, the sensors used for the localization function, the intended or first field of application, the system architecture of the localization function or sensor data fusion (e.g., filtering or snapshot methods) and the utilized integrity measures (e.g., PL and/or AL) are used as distinguishing properties.

For an application in aviation, several integrity algorithms are developed for localization functions focusing on the position solution. Initially, a single-frequency single-constellation GNSS receiver is utilized as only sensor and the system architecture implements a least-squares algorithm [25]. The integrity algorithms are based most often on Receiver Autonomous Integrity Monitoring (RAIM), which is using the overdetermined position solution computed by the mentioned snapshot method from the GNSS pseudorange observations (redundant measurements) to perform consistency checks [26]. Additionally, Detection, Identification and Adaption (DIA) procedures are applied, which include a global test to detect outlier presence assuming a certain statistical distribution and, depending on the implementation, a local test to identify them [20].

As GNSS develop further and integrity requirements become stricter, drawbacks of the first RAIM algorithms are identified, which include the general assumption of only one present outlier per epoch and the utilization of a single-frequency, single-constellation GNSS receiver [27,28]. This leads to further developments and extensions of RAIM: Several studies extend the concept of RAIM to multiple outliers, e.g., by Tu et al. [27]. Another development to modify RAIM is carried out by the Stanford GPS Lab. Blanch et al. [29] present a baseline version of Advanced Receiver Autonomous Integrity Monitoring (ARAIM), an extension of RAIM to multi-constellation dual-frequency GNSS. A modification to adapt the least-squares baseline version to a Kalman Filter (KF) version for Precise Point Positioning (PPP) is introduced by Gunning et al. [30]. Further developments published by the same author include an extension to integrate IMU observations into the localization function [31]. Pullen et al. [13] take the concept of ARAIM developed for aviation to automotive applications and describe the integration of ARAIM in the Globalstar Connected Car Program. Besides that, there are other extensions of RAIM to GNSS/IMU algorithms which focus on detecting outliers, e.g., for a tightly coupled fusion by Hewitson et al. [32] and Liu et al. [33]. In the latter reference, outliers are categorized in slowly/quickly growing or step errors and dedicated testing methods for each category are developed.

Another group of integrity algorithms is developed, especially for multi-sensor data fusion. Multiple studies for the different system architectures can be found in the literature, e.g., for detecting erroneous sensor data in a fusion using Bayesian method [34] and using integrity monitoring for KF applications [35]. A dedicated approach to compute PLs for a localization function used for automated driving is developed by GMV company and its partners. In the patent [36], Azaola Sàenz introduces the Isotropy-Based Protection Level (IBPL), which is designed for a localization function using GNSS pseudoranges and computes a PL based on least-squares residuals and scalar factors stemming from the Dilution of Precision (DOP), the number of observations and the number of estimated states. Building upon IBPL, an extension of this concept for system architectures based on a KF is developed, which is called Kalman Integrated Protection Level (KIPL). It is disclosed by Navarro Madrid in the patent [37]. For KIPL, the influence of all measurement inputs to the sensor data fusion are modeled dynamically and fused to a total error distribution which is used to compute the PL [38]. Results of KIPL in the ESCAPE research project are presented by Tijero et al. [39].

**Figure 2:**
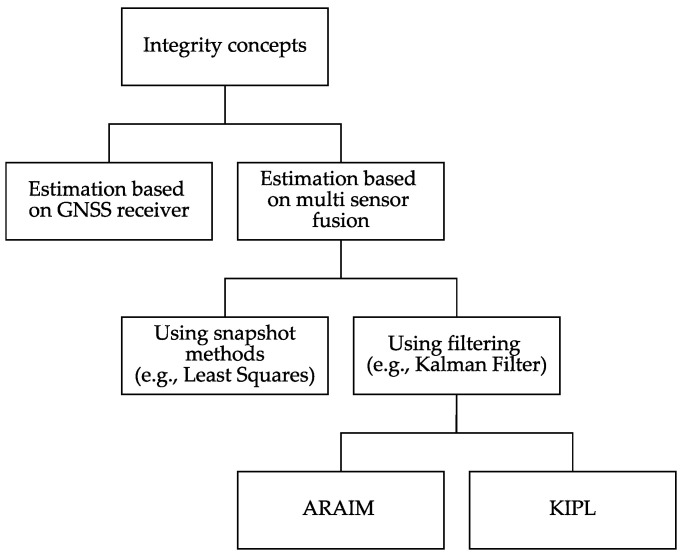
Decision tree for integrity concepts (representation not comprehensive).

Applying the mentioned grouping criteria to these integrity concepts from the literature is used to decide which one of them will serve as a basis for the implemented integrity algorithms. Figure 2 shows the resulting decision tree. Since the use case requires an integrity concept for a fusion algorithm using multiple sensors and filtering, ARAIM and KIPL are selected. To compare them to traditional approaches, an integrity concept based on the standard deviations estimated by the KF is selected as a third algorithm.

## 4. Implementation

In this Section, the three implemented integrity algorithms are explained. All of them provide a PL which is compared with the defined AL for the given use case. For the comparison, one of the fusion filters of the VDSE of UNICAR*agil* is used, which is explained in Section 4.1.

### 4.1. Fusion Algorithm

**Figure 3:**
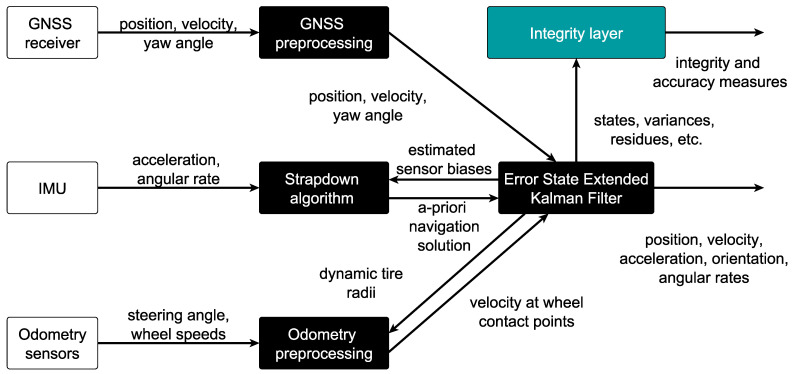
Block diagram of Global Navigation Satellite System (GNSS)/Inertial Measurement Unit (IMU)/odometry fusion filter used for comparison of implemented integrity algorithms (Figure based on [14]).

As mentioned in the introduction, one of the GNSS/IMU/odometry sensor fusion algorithms from the VDSE is used for the comparison of the implemented integrity algorithms. Figure 3 depicts the block diagram of the sensor data fusion in which an Error-State Extended Kalman Filter (ES-EKF) is implemented. The algorithm builds upon the developments and results of the PhD thesis of Reuper [40]. The main differences to Reuper’s PhD thesis [40] are the usage of a dual-antenna RTK GNSS receiver in a loose coupling and the integrity layer. Since the implemented equations for the ES-EKF [40] and the loose coupling of GNSS and IMU [19] are state of the art, they are omitted here but can be found in the cited literature.

The ES-EKF uses 19 states which are depicted in Table 1. Measurement updates from the GNSS observations (position, velocity, yaw angle) and from the odometry observations (velocity at wheel contact points) are used in the ES-EKF to estimate these states, while the IMU is processed in a strapdown algorithm utilizing the estimated sensor biases. Additionally, Zero Velocity Update (ZVU) and Zero Angular Rate Update (ZARU) are implemented as explained by Groves ([19] chapter 15.3) and applied when a standstill of the vehicle is detected by the odometry sensors, meaning that, for more than 0.5 s, all odometry sensors observe a velocity of zero.

Outputs of the filter are position, velocity, attitude and angular rate in three dimensions. Besides that, the integrity layer computes PLs, which in this comparison, is performed by one of the selected integrity algorithms explained in the following subsection.

Different quantities from the ES-EKF are used as inputs to the integrity algorithms. These include the estimated state covariance matrix *P* or the respective estimated standard deviations for the traditional approach. Additionally, KIPL utilizes the measurement matrices $H_{m}$, measurement noise covariance matrices $R_{{\mathrm{meas}}_{m}}$ and measurement residual vectors $y_{m}$ for all used measurement types *m* and the transition matrix *F*, as well as the estimated state *x*.

### 4.2. Integrity Algorithms

Each of the implemented integrity algorithms is given a name according to the underlying formulas or their basis from the literature. We introduced the concepts of the three integrity algorithms in a conference paper [14]. In this work, a complete implementation and further developments for more challenging GNSS reception conditions are presented. Many known integrity algorithms output over-optimistic PLs in difficult environmental conditions. The developments presented in this work aim to avoid that by amendments to our previously presented concepts [14]. The parameters were empirically tuned using the Stanford integrity diagram such that the specified performance criteria, especially regarding the IR, are fulfilled.

#### 4.2.1. kSigma

The first integrity algorithm represents the traditional approach of computing confidence levels based on the estimated standard deviations by the ES-EKF. The PLs are computed by scaling the respective standard deviations with a scalar factor *k*. Therefore, in the following, this algorithm is referred to as kSigma.

The equations for the horizontal and vertical position PL, $P\;L_{\mathrm{kSigma},\mathrm{pos}H}$ and $P\;L_{\mathrm{kSigma},\mathrm{pos}V}$, as well as for the heading PL, $P\;L_{\mathrm{kSigma},\psi }$, are given by

(1) $$\begin{array}{ccc} P\;L_{\mathrm{kSigma},\mathrm{pos}H} & = & k_{\mathrm{pos}H}\;\sigma_{H} \end{array}$$

(2) $$\begin{array}{ccc} P\;L_{\mathrm{kSigma},\mathrm{pos}V} & = & k_{\mathrm{pos}V}\;\sigma_{U} \end{array}$$

(3) $$\begin{array}{ccc} P\;L_{\mathrm{kSigma},\psi } & = & k_{\psi }\;\sigma_{\psi } \end{array}$$

with

(4) $$\sigma_{H}=\sqrt{\frac{\sigma_{E}^{2}+\sigma_{N}^{2}}{2}+\sqrt{{\left(\frac{\sigma_{E}^{2}+\sigma_{N}^{2}}{2}\right)}^{2}+\sigma_{EN}^{2}}}$$

where the variances of the east, north, up component and the east-north covariance of the position solution and the yaw standard deviation are expressed as $\sigma_{E}^{2}$, $\sigma_{N}^{2}$, $\sigma_{U}^{2}$, $\sigma_{EN}$, $\sigma_{\psi }$ respectively [41]. The scalar factors $k_{H}$ and $k_{V}$ are both set to three, representing an integrity risk of approximately 0.3%, assuming normally distributed errors. For $k_{\psi }$ an empirically defined factor of nine was chosen to not exceed the specified IR. In order to prevent over-optimistic PLs, lower bounds for the estimated standard deviations are introduced. A lower bound of 0.03 m for $\sigma_{H}$ and $\sigma_{U}$ as well as $0.017{}^{\circ }$ for $\sigma_{\psi }$ is applied before the PLs of Equations (1)–(3) are computed.

#### 4.2.2. Kalman Integrated Protection Level (KIPL)

While the first integrity algorithm is rather simple and straight forward in its equations, the second algorithm uses a more sophisticated approach including an implementation of the previously mentioned KIPL method, wherefore it is referred to as KIPL in the following. All explained computations for this integrity algorithm are performed analogously for the position and heading PL and differences in the parameters for the respective cases are marked with a subscript (pos or ψ).

In this algorithm, the estimation error of the ES-EKF is modeled as the sum of its contributions, meaning the measurement inputs to the filter [42]. These contributions are categorized by measurement type, whereby each measurement type *m* is modeled by a zero-mean multivariate Student distribution $t_{N_{m}}\left(R_{m}\right)$ [38]. In the use case of this work, filter one of the VDSE, there are six measurement types, namely, the GNSS position, GNSS velocity, GNSS heading angle and odometry velocity observations, as well as the zero updates, ZVU and ZARU. The PL is computed for the three-dimensional position and for the one-dimensional yaw angle, leading to $d_{\mathrm{pos}}=3$ and $d_{\psi }=1$. After the measurement update step of the ES-EKF, the distribution $t_{N_{m}}\left(R_{m}\right)$ of the respective measurement type *m* is updated by recalculating it as the sum of two zero-mean multivariate Student distributions $t_{N_{m}}\left(R_{m1}\right)$ and $t_{N_{m}}\left(R_{m2}\right)$ which model the estimation errors of the filter in the prediction and measurement update step respectively [38]. In the patent [37] the Equations (5)–(13) to calculate $N_{m1}$, $R_{m1}$, $N_{m2}$ and $R_{m2}$ are given with the measurement matrix $H_{m}$, the state covariance matrix *P*, the measurement noise covariance matrix $R_{{\mathrm{meas}}_{m}}$, the measurement residual vector $y_{m}$, the transition matrix *F* from the ES-EKF and the tuning parameters β and $\rho_{m}$.

(5) $$\begin{array}{c} S_{m}=H_{m}PH_{m}^{T}+R_{{\mathrm{meas}}_{m}}, \end{array}$$

(6) $$\begin{array}{c} K_{m}=PH_{m}^{T}S_{m}^{-1}, \end{array}$$

(7) $$\begin{array}{c} N_{m1}=n_{m}+\beta \;N_{m1}^{\prime }, \end{array}$$

(8) $$\begin{array}{c} n_{m}=n_{{\mathrm{obs}}_{m}}-tr\left(H_{m}K_{m}\right)-tr\left(\rho_{m}H_{m}K_{m}\right), \end{array}$$

(9) $$\begin{array}{c} R_{m1}=r_{m}^{2}K_{m}S_{m}K_{m}^{T}, \end{array}$$

(10) $$\begin{array}{c} r_{m}^{2}=\left(y_{m}^{T}S_{m}^{-1}y_{m}+\beta N_{m1}^{\prime }r_{m}^{\prime }\right)\;\frac{1}{N_{m1}} \end{array}$$

(11) $$\begin{array}{c} N_{m2}=N_{m}^{\prime }, \end{array}$$

(12) $$\begin{array}{c} R_{m2}=UR_{m}^{\prime }U^{T}, \end{array}$$

(13) $$\begin{array}{c} U=\left(I-\sum_{m}K_{m}H_{m}\right)\;F \end{array}$$

Table 2 shows the initial values required for these Equations. In general, a transpose of a matrix *A* is represented by $A^{T}$, while $A^{\prime }$ is the value of matrix *A* from the previous step and *I* stands for the identity matrix.

Having $t_{N_{m}}\left(R_{m1}\right)$ and $t_{N_{m}}\left(R_{m2}\right)$ computed, in the next step, their sum is approximated by using

(14) $$R_{m}=R_{m1}+R_{m2}+D_{m}+D_{m}^{T}$$

with

(15) $$D_{m}=r_{m}K_{m}S_{m}^{-1/2}A_{m}^{\prime }U^{\prime }$$

and solving

(16) $$\begin{array}{cc} \; & N_{m}^{\frac{d-2}{2}}t_{m}^{2-d}{\left(1+t_{m}^{-2}\right)}^{-\frac{N_{m}+2-2}{2}}=\\ & N_{m1}^{\frac{d-2}{2}}t_{m1}^{N_{m1}}exp\left\{\frac{\left(N_{m1}+N_{m2}\right)N_{m1}}{2N_{m2}}t_{m2}^{2}\right\}+N_{m2}^{\frac{d-2}{2}}t_{m2}^{N_{m2}}exp\left\{\frac{\left(N_{m1}+N_{m2}\right)N_{m2}}{2N_{m1}}t_{m1}^{2}\right\} \end{array}$$

numerically for $N_{m}$ with

(17) $$\begin{array}{c} t_{m1}={\left[\frac{N_{m1}tr\left(R_{m1}\right)}{tr(S)}\right]}^{\frac{1}{2}}, \end{array}$$

(18) $$\begin{array}{c} t_{m2}={\left[\frac{N_{m2}tr\left(R_{m2}\right)}{tr(S)}\right]}^{\frac{1}{2}}, \end{array}$$

(19) $$\begin{array}{c} t_{m}={\left[\frac{N_{m}tr\left(R_{m}\right)}{tr(S)}\right]}^{\frac{1}{2}}, \end{array}$$

(20) $$\begin{array}{c} S=\left(1+\omega \right)\;\left(N_{m1}R_{m1}+N_{m2}R_{m2}\right) \end{array}$$

and the tuning parameter ω [38]. In a third step, the update of $A_{m}$ is calculated as disclosed in the patent [37] by

(21) $$A_{m}=r_{m}\rho_{m}S_{m}^{-1/2}K_{m}^{\prime }+\rho_{m}A_{m}^{\prime }U^{\prime }.$$

The PL results as sum of the error bounds $B_{m}$ from each measurement type *m*

(22) $$P\;L_{\mathrm{KIPL}}=\sum_{m}B_{m}$$

using

(23) $$B_{m}=k\left(\alpha ,N_{m}\right)\;b_{m}$$

with

(24) $$b_{m}={\left(tr_{m}/d\right)}^{1/2},$$

where $tr_{m}$ represents the trace of the matrix $R_{m}$ over the states for which the PL is computed for, meaning the three PE states in case of the position PL and the HE state in case of the heading PL, and solving

(25) $$\frac{2}{B\left(\frac{2}{d},\frac{N_{m}}{2}\right)}\int_{k}^{\infty }\frac{y^{d-1}}{{\left(1+y^{2}\right)}^{(N_{m}+d)/2}}dy=\alpha$$

numerically for $k(\alpha ,N_{m})$ with the integrity risk α=0.01 and the beta function B(x,y) [37].

For the six mentioned measurement types, the parameters used in this implementation are depicted in Table 3, wherein $I_{n}$ represents the identity matrix of order *n*. Out of these parameters, ω and β are empirically found tuning parameters and $\rho_{m}$ represents a correlation factor [37]. The parameter ω has to be greater than one and a value of ten represents high-confidence levels, according to Welte [38]. The value range of β is given as 0≤β<1 and a non-zero value represents the influence of statistics of previous epochs on the characterization of the error noise at the present epoch [37]. When tuning the parameters for the used setup of sensors and fusion filter, the goal was to minimize the outputted PLs, while not exceeding the specified IR.

The number of observations in a measurement type determines $n_{{\mathrm{obs}}_{m}}$, leading to three for the three-dimensional GNSS position and velocity solution, and to one for the GNSS yaw solution. For the odometry, only the horizontal velocity is considered and only one lateral velocity per axle, as described by Reuper [40]. Therefore, there are six odometry observations. The detection of vehicle standstill for ZVU and ZARU is considered as one observation.

Motivated by the results from experiments with real measurement data during the implementation, an empirical factor of two is applied to the position PL from Equation (22). Eventually, the position PL is multiplied by $\sqrt{2}$ to obtain the 2D horizontal $P\;L_{\mathrm{KIPL},\mathrm{posH}}$ from the 1D $P\;L_{\mathrm{KIPL},\mathrm{pos}}$. Note that the PL stays constant in-between measurement updates. Not updated error bounds $B_{m}$ are taken from the previous step.

Additionally, two empirically motivated measures are introduced to prevent over-optimistic PLs for $P\;L_{\mathrm{KIPL},\mathrm{posH}}$ and $P\;L_{\mathrm{KIPL},\psi }$. These are a dynamic buffer to account for high dynamics and a dynamic lower bound to take difficult GNSS reception conditions into account.

For the lower bound $B_{\mathrm{low}}$, a quadratic function in the form

(26) $$B_{\mathrm{low}}\left(q_{\mathrm{cnt}}\right)=a_{2}\;q_{\mathrm{cnt}}^{2}+a_{1}\;q_{\mathrm{cnt}}+a_{0}$$

is used, with the counter $q_{\mathrm{cnt}}$ and the parameters $a_{0}$, $a_{1}$, $a_{0}$ which are depicted in Table 4.

The error in the position with no GNSS reception grows approximately like a quadratic function with respect to time, which motivates this design choice. Since the heading error grows approximately linearly with respect to time, the parameter $a_{2}$ is set to zero for the heading lower bound. The lower bound is applied in two kinds of situations with respect to the GNSS reception conditions, leading to two lower bounds $B_{\mathrm{low},\;\mathrm{noGNSS}}\left(q_{\mathrm{cnt},\;\mathrm{noGNSS}}\right)$ and $B_{\mathrm{low},\;\mathrm{noRTK}}\left(q_{\mathrm{cnt},\;\mathrm{noRTK}}\right)$, which each have a separate counter. The counters are named $q_{\mathrm{cnt},\;\mathrm{noGNSS}}$ and $q_{\mathrm{cnt},\;\mathrm{noRTK}}$, count time in seconds and are both initialized with zero. If there is no GNSS reception for one epoch, meaning the GNSS receiver does not output a position solution to the loosely coupled filter, the counter $q_{\mathrm{cnt},\;\mathrm{noGNSS}}$ will start counting. It continues counting until there is GNSS-RTK reception for a time $q_{\mathrm{reset}}$ (given in Table 4), which leads to a reset of the counter $q_{\mathrm{cnt},\;\mathrm{noGNSS}}$ to zero. The counter $q_{\mathrm{cnt},\;\mathrm{noRTK}}$ is activated if $q_{\mathrm{cnt},\;\mathrm{noGNSS}}$ is equal to zero and is counting the time since the last GNSS-RTK reception, but will only be used if it is higher than the threshold $q_{\mathrm{reset}}$. As soon as there is an epoch with GNSS-RTK reception, the counter $q_{\mathrm{cnt},\;\mathrm{noRTK}}$ will be reset to zero. Eventually, the sum of the two lower bounds $B_{\mathrm{low},\;\mathrm{noGNSS}}\left(q_{\mathrm{cnt},\;\mathrm{noGNSS}}\right)$ and $B_{\mathrm{low},\;\mathrm{noRTK}}\left(q_{\mathrm{cnt},\;\mathrm{noRTK}}\right)$ is applied to $P\;L_{\mathrm{KIPL},\mathrm{posH}}$ and $P\;L_{\mathrm{KIPL},\psi }$ respectively, which will be explained with formulas in the following.

The buffer $B_{\mathrm{buffer},\mathrm{posH}}$ is applied after the lower bound and computed as

(27) $$B_{\mathrm{buffer},\mathrm{posH}}=k_{\mathrm{posH},\mathrm{buffer}}\;a_{\mathrm{filtered},H}$$

with the horizontal acceleration $a_{\mathrm{filtered},H}$ as a moving average from the past five seconds and a scalar factor $k_{\mathrm{posH},\mathrm{buffer}}$ of 0.05. Since in the experiments, the influence of high dynamics was only observed on the position error, the dynamic buffer is only applied to the position PL and not to the heading PL.

**Figure 4:**
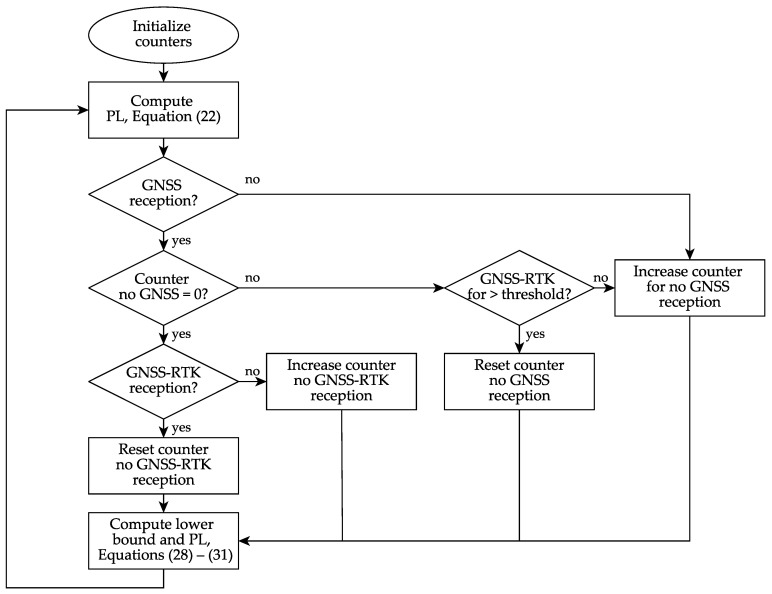
Flow chart of KIPL integrity algorithm PL computation.

Figure 4 summarizes the described procedure to compute PLs for the KIPL integrity algorithm and concludes with the following equation

(28) $$\begin{array}{cc} P\;L_{\mathrm{KIPL},\mathrm{posH},\mathrm{mod}}= & \left\{\begin{array}{cc} P\;L_{\mathrm{KIPL},\mathrm{posH}}+B_{\mathrm{buffer},\mathrm{posH}} & P\;L_{\mathrm{KIPL},\mathrm{posH}}\geq B_{\mathrm{low},\mathrm{posH}}\\ B_{\mathrm{low},\mathrm{posH}}+B_{\mathrm{buffer},\mathrm{posH}} & P\;L_{\mathrm{KIPL},\mathrm{posH}}<B_{\mathrm{low},\mathrm{posH}} \end{array}\right. \end{array}$$

(29) $$\begin{array}{cc} P\;L_{\mathrm{KIPL},\psi ,\mathrm{mod}}= & \left\{\begin{array}{cc} P\;L_{\mathrm{KIPL},\psi } & \;\;\;\;\;\;\;\;\;\;\;P\;L_{\mathrm{KIPL},\psi }\geq B_{\mathrm{low},\psi }\\ B_{\mathrm{low},\psi } & \;\;\;\;\;\;\;\;\;\;\;P\;L_{\mathrm{KIPL},\psi }<B_{\mathrm{low},\psi } \end{array}\right. \end{array}$$

using

(30) $$\begin{array}{cc} B_{\mathrm{low},\mathrm{posH}}= & \left\{\begin{array}{cc} B_{\mathrm{low},\mathrm{posH},\mathrm{noGNSS}}+B_{\mathrm{low},\mathrm{posH},\mathrm{noRTK}} & q_{\mathrm{cnt},\mathrm{noRTK}}\geq q_{\mathrm{reset}}\\ B_{\mathrm{low},\mathrm{posH},\mathrm{noGNSS}} & q_{\mathrm{cnt},\mathrm{noRTK}}<q_{\mathrm{reset}} \end{array}\right. \end{array}$$

(31) $$\begin{array}{cc} B_{\mathrm{low},\psi }= & \left\{\begin{array}{cc} B_{\mathrm{low},\psi ,\mathrm{noGNSS}}+B_{\mathrm{low},\psi ,\mathrm{noRTK}} & \;\;\;\;q_{\mathrm{cnt},\mathrm{noRTK}}\geq q_{\mathrm{reset}}\\ B_{\mathrm{low},\psi ,\mathrm{noGNSS}} & \;\;\;\;q_{\mathrm{cnt},\mathrm{noRTK}}<q_{\mathrm{reset}} \end{array}\right.. \end{array}$$

#### 4.2.3. Advanced Receiver Autonomous Integrity Monitoring (ARAIM)

The third integrity algorithm includes an implementation of ARAIM introduced by the Stanford GPS Lab as it was presented by Gunning et al. [30] for PPP using Multiple Hypothesis Solution Separation (MHSS). To apply the mentioned implementation to the given use case, RTK solutions as one-out subsets, meaning solutions with all but one of the satellites in sight, and the all-in-view solution are computed. These computations are performed with version demo5_b33c of RTKLIB provided by Everett [43], which is based on the original version of RTKLIB by Takasu [44]. GNSS correction data, meaning ultra rapid orbit and clock corrections (clk and sp3 files), are downloaded from the GeoForschungsZentrum (GFZ) Potsdam. The broadcast ephemerids from the German Federal Agency for Cartography and Geodesy (BKG) are used. For the following fusion, the GNSS position solution and estimated standard deviation computed with the mentioned version of RTKLIB are combined with the yaw (only for filter initialization) and velocity values of the real-time solution provided by the RTK-GNSS receiver. Afterwards, the previously described fusion filter is applied for all subsets and the all-in-view solution. Analogously to kSigma, a constant lower bound for the estimated standard deviation for the position solution of 0.005 m is applied before it is multiplied by an empirical factor of three.

With the equations given by Gunning et al. [30], the PL level is computed by

(32) $$P\;L_{\mathrm{ARAIM}}=\max_{i}\left(T_{i}+Q^{-1}\left(\frac{P\;H\;M\;I\;}{N\;P(H_{i})}\right)\sigma^{\left(i\right)}\right)$$

with the threshold

(33) $$T_{i}=Q^{-1}\left(\alpha_{i}P_{\mathrm{FA}}\right)\sigma_{\mathrm{ss}}^{\left(i\right)}$$

using

(34) $$\sigma_{\mathrm{ss}}^{(i)2}=\sigma^{(i)2}-\sigma^{(0)2},$$

the standard deviation for the *i*th subset $\sigma^{\left(i\right)}$ and for the all-in-view solution $\sigma^{\left(0\right)}$, the number of subsets *N*, the complement of the normal cumulative distribution function *Q* and its inverse $Q^{-1}$. The parameters are also taken from Gunning et al. [30], as $\alpha_{i}=\frac{1}{N}$, $P\;H\;M\;I\;=\frac{1}{3}10^{-7}$, $P_{\mathrm{FA}}=\frac{1}{3}10^{-6}$, $P\left(H_{i}\right)=10^{-5}$. Eventually, the PL for the HE is already given as $P\;L_{\mathrm{ARAIM},\psi }$ and for the horizontal PE results as

(35) $$\begin{array}{cc} P\;L_{\mathrm{ARAIM},\mathrm{posH}}= & \sqrt{P\;L_{\mathrm{ARAIM},E}^{2}+P\;L_{\mathrm{ARAIM},N}^{2}} \end{array}$$

with the PL’s components in east and north direction, $P\;L_{\mathrm{ARAIM},E}$ and $P\;L_{\mathrm{ARAIM},N}$, from Equation (32).

In order to account for difficult GNSS reception conditions, additional measures are taken. The RTKLIB quality level is used as an indication of these conditions. Only a quality level of one means that the integer ambiguity for the RTK-GNSS solution is solved properly [45]. If RTKLIB outputs a non-ideal quality level (meaning non-equal to one), an empirical factor of three is used for both PLs, $P\;L_{\mathrm{ARAIM},posH}$ and $P\;L_{\mathrm{ARAIM},\psi }$. Besides that, a constant value of $0.1{}^{\circ }$ is added to $P\;L_{\mathrm{ARAIM},\psi }$.

## 5. Experiments and Results

For the comparison and evaluation of the implemented integrity algorithms for the VDSE in UNICAR*agil*, four measurement experiments are used, which represent four driving scenarios with different environmental conditions. Before these are explained in detail (Section 5.2), the sensor setup is described (Section 5.1). Afterwards, the results are evaluated (Section 5.3).

### 5.1. Sensor Setup

Table 5 depicts the sensors and its properties used for the previously explained sensor data fusion filter of the VDSE, as well as for the reference solution.

The reference solution is obtained from a post-processing evaluation of the observations from a navigation grade Ring Laser Gyroscope (RLG)-IMU (iMAR Navigation GmbH, St. Ingbert, Germany) and a multi-frequency, multi-constellation GNSS receiver (NovAtel Inc., Calgary, AB, Canada) in the software NovAtel WayPoint Inertial Explorer 8.90 (NovAtel Inc., Calgary, AB, Canada). All measurement experiments are carried out with a conventional vehicle (Volkswagen T5), since the prototype vehicles in UNICAR*agil* are still under construction. The lever arms between the sensors were determined by a photogrammetric survey of the vehicle.

### 5.2. Experiments

The four selected measurement experiments are extracted from three data sets that we recorded in May, 2019 with the measurement vehicle of the Chair of Physical and Satellite Geodesy at Technical University of Darmstadt, a Volkswagen T5 equipped with the mentioned sensors from Table 5. Table 6 summarizes the experiments’ key information.

A method developed within the project UNICAR*agil* [46] is utilized to identify potential causal factors for integrity failure of the VDSE by using Fault Tree Analysis (FTA) and System Theoretic Process Analysis (STPA) [47]. The selection of measurement experiments is made such that the selected scenarios include as many of the identified potential causal factors as possible in real measurements. The selected scenarios represent four typical driving scenarios for automated vehicles and they contain a variety of different challenges regarding the GNSS reception conditions. The difficulty for the VDSE and the integrity algorithms increases steadily from the scenario on the airfield as ideal scenario with no difficulties regarding the GNSS reception conditions to the urban scenario with numerous challenges including signal obstruction, multipath and Non-Line-of-Sight (NLOS) reception.

**Figure 5:**
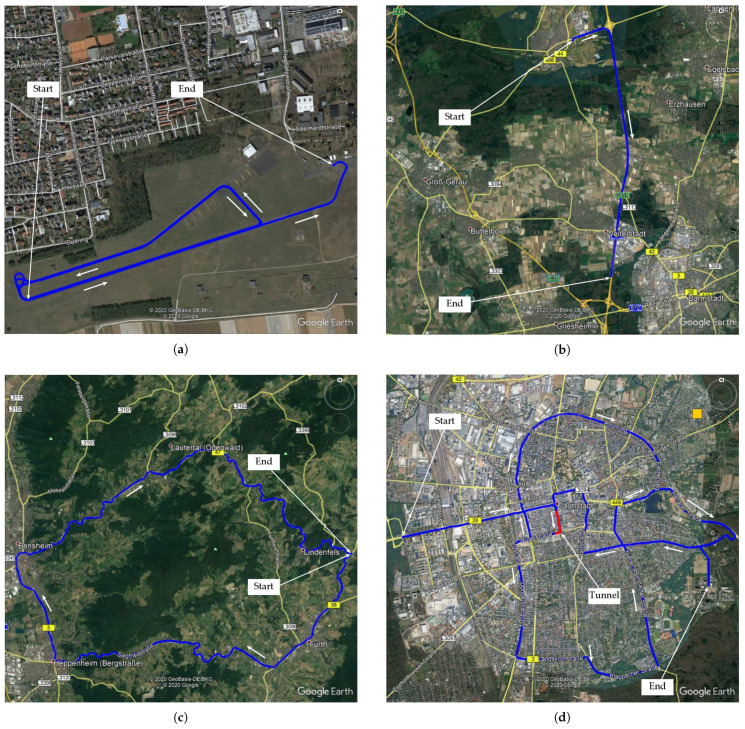
Driven trajectory in the four selected experiments: (**a**) TU Darmstadt airfield Griesheim, Germany, (**b**) Highway A5 near Darmstadt, Germany, (**c**) Odenwald near Heppenheim, Germany, (**d**) Darmstadt, Germany (Screenshots taken from Google Earth Pro, map credits given on the screenshot).

The first experiment stems from a data set recorded on the Technical University of Darmstadt’s airfield (August-Euler-Flugplatz) in Griesheim, Germany on 7 May 2019. On the airfield, there is open sky view and the GNSS receptions conditions are ideal. Two and a half rounds over the runway and taxiway are driven, first anti-clockwise then clockwise. Figure 5a depicts the trajectory in a satellite picture.

In the second experiment, the GNSS reception conditions are not ideal anymore. It was recorded on 9 May 2019 between Mörfelden and Darmstadt, Germany, mainly on highway A5 direction south. The trajectory is depicted in Figure 5b and contains frequent signal obstruction, due to e.g., bridges and overhead road sign structures.

The third data set stems form a measurement recorded on 6 May 2019 near Heppenheim, Germany. The driven trajectory is shown in Figure 5c and leads through the Odenwald, a mountain range with forest areas. The route contains driving on country roads which leads to more difficult GNSS reception conditions due to signal obstruction from vegetation in the forest and buildings in the towns.

The fourth experiment was recorded in the city center of Darmstadt, Germany on 7 May 2019. The GNSS reception conditions are degraded since the route contains GNSS signal obstruction due to multi-level buildings and due to vegetation while passing a forest as well as due to total signal blockage in a tunnel in Darmstadt downtown which is passed through twice (Figure 5d).

### 5.3. Results

**Figure 6:**
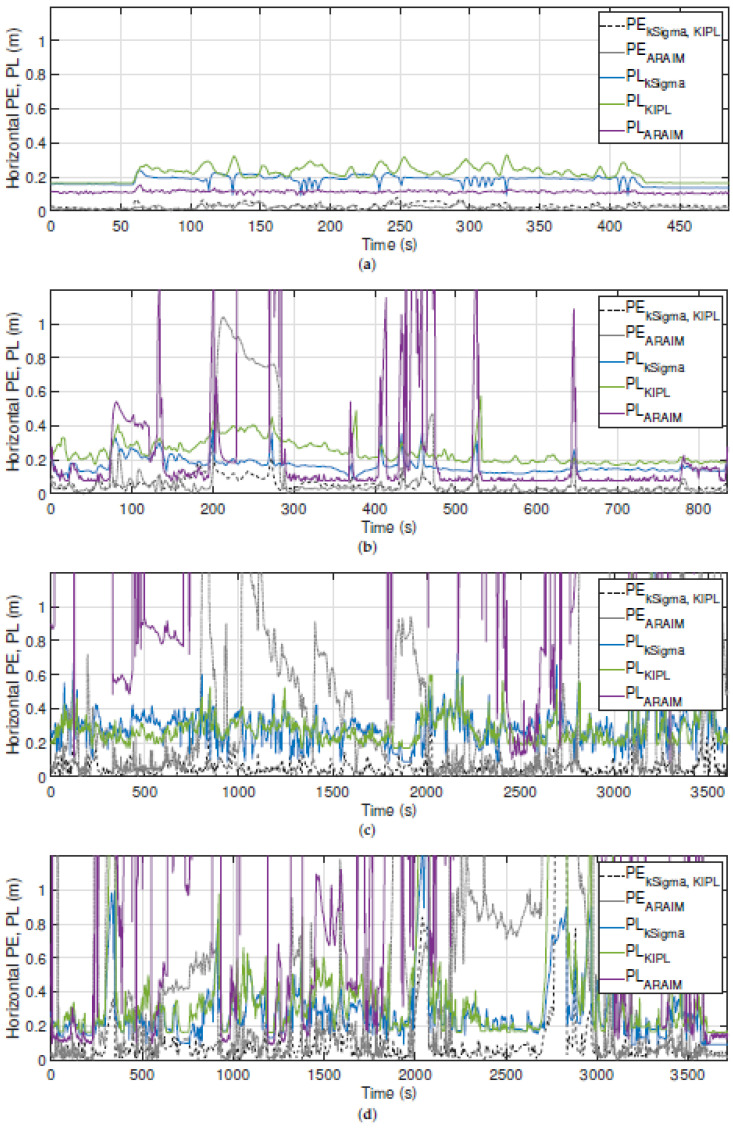
Comparison of the horizontal position error and the three implemented protection levels in the four selected scenarios: (**a**) airfield, (**b**) highway, (**c**) country road and (**d**) urban.

In order to evaluate the performance of the implemented PLs, first the position PLs are analyzed before a closer look at the heading PLs is taken. Figure 6 depicts the position PLs of the three implemented integrity algorithms over time in the four described experiments as solid lines. Besides that, also the respective error (PE) is plotted as a dashed line, which is computed as difference between the reference solution and the fusion result. This leads to different PEs for kSigma and KIPL on one side and ARAIM on the other side, since for ARAIM another GNSS solution is used. As explained in Section 4.2.3, ARAIM uses the GNSS solution of RTKLIB, while the other two integrity algorithms rely on the real-time solution of the RTK-GNSS receiver, which leads to different fusion results and therefore to different PEs. For the ordinate, a maximum value of 1.2 m is chosen to focus on the critical area for the integrity performance, meaning around the chosen AL of 0.6 m.

There are several differences between the integrity algorithms with respect to the performance of the provided PLs observable. Most notably, the PL range changes dramatically between the scenarios. While on the airfield, all PLs stay under the defined AL of 0.6 m, this is not the case anymore in more challenging GNSS reception conditions.

In general, the more challenging the conditions are, the higher the PLs are, which seems plausible but leads to lower availability in challenging GNSS reception conditions. Especially, the chosen implementation of ARAIM is strongly effected by GNSS signal obstruction, e.g., by bridges and overhead traffic sign structures on the highway. These are the root cause for the peaks in $P\;L_{\mathrm{ARAIM}}$ during this scenario, which results in a lower availability for ARAIM. On country roads and in urban areas, $P\;L_{\mathrm{ARAIM}}$ is also significantly higher than the other two PLs.

Besides that, in the four scenarios, the PL of KIPL is slightly greater than the one provided by kSigma, but both PLs stay mostly under the defined AL. When analyzing the relationship between PL and PE, KIPL seems to provide the most reliable PL out of the three implemented PLs, since the other two show some weaknesses in more challenging conditions. This becomes observable, e.g., in the highway scenario at around 220 s for ARAIM and in the urban scenario at around 2750 s for kSigma.

These qualitative observations are confirmed by the quantitative results. Table 7 depicts the percentage of epochs in which the provided position PL is binding the PE. According to the specified IR of 1%, this value should not be lower than 100%−1%=99%. In ideal GNSS reception conditions, like on the airfield, all integrity algorithms can keep this specification. With more difficult circumstances, this is not the case anymore, and in the urban scenario, only the KIPL integrity algorithm reaches a value of more than 99% of the epochs with PE<PL.

In Table 8, the availability of the fusion filter with the implemented integrity algorithms in the four scenarios is depicted. As explained in Section 2.1, the filter becomes unavailable if the provided PL exceeds the specified AL. As anticipated by the qualitative analysis, the availability decreases in challenging GNSS reception conditions, especially for the chosen implementation of ARAIM. The availability of the fusion filter with kSigma and KIPL is very similiar with a slight advantage for kSigma.

All in all, KIPL shows the best performance for the position PL in the chosen implementation and with the selected tuning parameters in the four selected experiments of this work. Therefore, only for KIPL, the detailed analysis for the four experiments utilizing Stanford integrity diagrams is conducted, now also including the heading PL.

**Figure 7:**
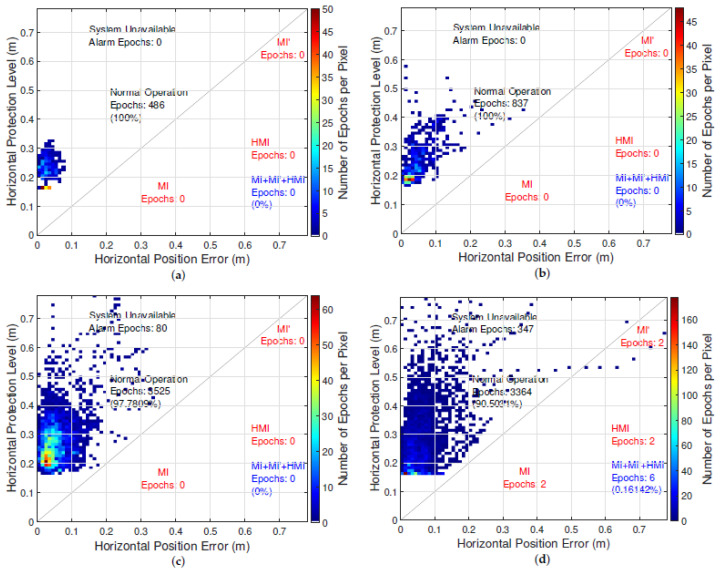
Stanford integrity diagrams for the protection level of the horizontal position error computed with chosen implementation of KIPL integrity algorithm in the four selected scenarios: (**a**) airfield, (**b**) highway, (**c**) country road and (**d**) urban.

**Figure 8:**
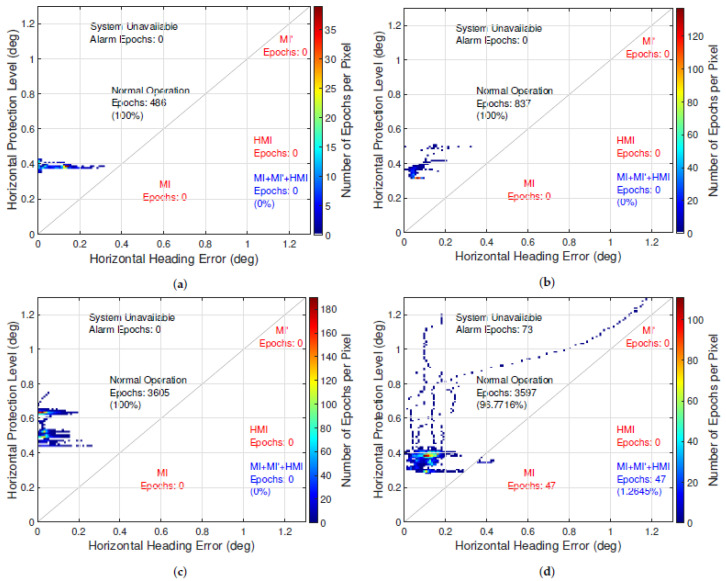
Stanford integrity diagrams for the protection level of the heading error computed with chosen implementation of KIPL integrity algorithm in the four selected scenarios: (**a**) airfield, (**b**) highway, (**c**) country road and (**d**) urban.

Figure 7 and Figure 8 depict the Stanford Integrity Diagrams for the four selected scenarios for the position and heading PL of the chosen implementation of KIPL. The previously mentioned correlation of GNSS reception conditions and magnitude of the position PL becomes more visible in Figure 7. For the heading PL, the same trend can be observed in Figure 8. Regarding the performance in terms of integrity and availability, the results for position and heading PL of KIPL are also similar.

In ideal and good GNSS reception conditions (airfield and highway scenario), the PLs bound the errors at all times and stay under the defined ALs, which leads to an availability of 100%.

In the country road scenario, for position and heading, the PLs also bound the errors at all times. In case of the position PL, it exceeds the chosen AL in 2.2% of the epochs, while for the heading solution, there is an availability of 100% in this scenario. For both PLs, no misleading or hazardous operation events are occurring.

Only in the very challenging GNSS reception conditions of the urban environment in the fourth chosen scenario does KIPL output misleading information. For the position PL, this is the case in less than 0.2% of the epochs in this scenario, which is lower than the specified IR of 1%. For the heading PL in this experiment, the specified integrity risk is slightly exceeded: In 1.3% of the epochs the HE exceeds the provided heading PL. The availability of the heading solution is slightly higher than for the position solution in the chosen implementation of KIPL, which leads to 100% availability in three of the four scenarios (two out of four for the position solution) and about 96% availability of the heading solution (about 90% for the position solution) in the urban scenario.

Conclusively, the chosen implementation of KIPL with the selected tuning parameters shows a better performance than the other two implemented integrity algorithms in the four selected experiments. The specified IR can be kept in all scenarios, except for the heading PL in the very challenging urban environment where it exceeds it slightly. The position and heading solution are available at all times in good GNSS reception conditions with this integrity algorithm implementation. In challenging GNSS reception conditions, the availability decreases to about 96% for rural areas and about 90% for urban areas, respectively.

## 6. Conclusions and Outlook

In this work, we developed and implemented three integrity algorithms for sensor data fusion algorithms to estimate the vehicle’s dynamic state for an application in a research vehicle for automated driving. Requirements for the integrity algorithms were derived from the literature and known integrity concepts were reviewed. While many other integrity concepts from the literature are only applicable to sensor fusion by snapshot methods, the three implemented integrity algorithms are compatible with multi-sensor fusion in a ES-EKF like it is used in the VDSE of the research project UNICAR*agil* and output a PL for the position and heading (yaw angle) solution.

Four experiments with data from real driving measurements were recorded and used to compare and evaluate the integrity algorithm’s performance. A special focus is on the GNSS reception conditions, which have a strong influence on the VDSE’s estimated state’s quality. In favorable to mixed GNSS reception conditions, like in the first three experiments, all implemented PLs bound the PE within the specified IR at all times. In the last experiment, in an urban environment, the GNSS reception conditions are more challenging, which results in a slightly higher IR than specified.

All in all, the integrity algorithm KIPL showed the best performance in the four experiments, when the chosen implementation and set of tuning parameters is used. In contrast to the other two implemented integrity algorithms, the specified IR is kept for the position PL in all four experiments and only slightly exceeded for the heading PL in the urban environment. The availability in the chosen experiments with the specified AL is 100% in favorable GNSS reception conditions and about 96% for rural areas and about 90% for urban areas, respectively.

The comparison shows that reliable integrity information for the position and heading solution can be provided by the implemented integrity algorithms. Even though all three integrity algorithms provide a PL, their concepts and underlaying formulas differ substantially. As mentioned before, the implemented PLs bound the PE in all experiments but their magnitude varies, which has a significant influence on the availability. When selecting an integrity algorithm for the specific application of a fusion algorithm in the VDSE in the research project UNICAR*agil*, this point and additional aspects like computation effort for a possible use in real-time will have to be taken into account.

In future work, the implemented integrity algorithms will be part of the integrity layer of the VDSE. This layer will serve as an input to a voting algorithm which generates a unique output of the VDSE out of the results from the different sensor fusion algorithms using subsets of the available sensor data in this service in UNICAR*agil*. To do so, the integrity algorithms will be extended to the whole vehicle’s state, including the velocity, acceleration and angular rate. While developing and testing the voting algorithm, the integrity layer’s performance will be continuously monitored, and further algorithm optimization and parameter tuning will be applied if necessary.

Another aspect of future work will be to develop a strategy of how to react to an integrity alarm of a fusion algorithm in the VDSE and how to recover the system to a safe operational state. This task will be solved together with other services in UNICAR*agil*, including the self-perception service, which is explained e.g., by Buchholz et al. [17].

## Figures and Tables

**Table 1:** States of error-state extended Kalman filter in fusion filter used for comparison of implemented integrity algorithms.

State	Dimension	Unit	Description
δΨ	3	rad	Misalignment
δv	3	m/s	Velocity error
δp	3	m	Position error
$\delta b_{\omega }$	3	rad/s	Gyroscope offset error
$\delta b_{a}$	3	m/s2	Accelerometer offset error
$\delta r_{d}$	4	m	Dynamic tire radius error

**Table 2:** Initial values for KIPL integrity algorithm (values based on [37]).

Parameter	Value
$N_{m}$	1
$N_{m1}$	1
$R_{m}$	0
$A_{m}$	0
$r_{m}$	0

**Table 3:** Parameters for Kalman Integrated Protection Level (KIPL) integrity algorithm.

Parameter	GNSS Position	GNSS Velocity	GNSS Heading	Odometry	ZVU	ZARU
ω	10	10	10	10	10	10
β	0.99	0.99	0.99	0.99	0.99	0.99
$n_{{\mathrm{obs}}_{m}}$	3	3	1	6	1	1
$\rho_{m}$	$0.9\;I_{3}$	$0.9\;I_{3}$	0.9	$0.9\;I_{6}$	0.9	0.9

**Table 4:** Parameters for dynamic lower bound for KIPL integrity algorithm.

Parameter	Position	Heading
$a_{2}$	$0.0003\;m/s^{2}$	0
$a_{1}$	0.035 m/s	0.013deg/s
$a_{0}$	0.075 m	0.05deg
$q_{\mathrm{reset}}$	5 s	5 s

**Table 5:** Sensors and processing of the fusion filter of the vehicle dynamic state estimation of UNICAR*agil* used to compare the implemented integrity algorithms and of the reference solution.

	Implemented Fusion Filter (VDSE)	Reference
Processing	Real-time capable	Post-processing
	(MATLAB code)	(NovAtel WayPoint Inertial Explorer 8.90)
IMU	Micro-Electro-Mechanical System	Ring Laser Gyroscope
	(Sensonor STIM300)	(iMAR iNAV-RQH1003)
GNSS	dual-frequency,	multi-frequency,
	multi-constellation,	multi-constellation,
	dual-antenna	single-antenna
	RTK-GNSS receiver	RTK-GNSS receiver
	(NovAtel OEM7720)	(NovAtel OEM729)
Odometry	production-line odometry	-
	(Volkswagen T5)	

**Table 6:** Characteristics of the four selected scenarios to compare the implemented integrity algorithms.

	Airfield	Highway	Country Road	Urban
GNSS recept- ion conditions	Ideal (open sky view)	Good (bridges and overhead sign structures)	Mixed (vegetation and small towns)	Challenging (downtown, passing twice a tunnel)
Location	TU Darmstadt airfield Griesheim, Germany	Highway A5 near Darmstadt, Germany	Odenwald near Heppenheim, Germany	Darmstadt, Germany
Duration	7 min	14 min	60 min	62 min
Date	7 May 2019	9 May 2019	6 May 2019	7 May 2019

**Table 7:** Percentage of epochs in which the error in the position solution of the sensor data fusion algorithm is bounded by the implemented protection levels.

Error < PL (% of Epochs)	Airfield	Highway	Country Road	Urban
Position-kSigma	100.0	100.0	99.8	96.1
Position-KIPL	100.0	100.0	100.0	99.8
Position-ARAIM	100.0	96.3	99.8	97.7

**Table 8:** Availability of the fusion filter with the implemented integrity algorithms.

PL < AL (% of Epochs)	Airfield	Highway	Country Road	Urban
Position-kSigma	100.0	100.0	97.6	90.8
Position-KIPL	100.0	100.0	97.8	90.5
Position-ARAIM	100.0	83.1	9.0	26.5

## Data Availability

The data presented in this study are available on request from the corresponding author.
